# The importance of perseverance, pilot studies and the search for effective adjuvant therapies in the management of tuberculous pericarditis

**Published:** 2016

**Authors:** Arthur Mutyaba, Mpiko Ntsekhe

**Affiliations:** Division of Cardiology, University of Cape Town Medical School, Cape Town, South Africa; Division of Cardiology, University of Cape Town Medical School, Cape Town, South Africa

Tuberculous pericarditis remains one of the most feared manifestations of extra-pulmonary tuberculosis (TB). The relatively high morbidity and mortality rates associated with the condition arise via two distinct mechanisms. The first is related to the combined impact of the virulence of Mycobacterium tuberculosis (MTb) itself and TB-induced dysregulated immune responses in both HIV-positive and -negative individuals, resulting in disseminated infection, multi-organ involvement, and prolonged acute infection.1 The second mechanism is related to compressive pericardial disease (cardiac tamponade, effusive constrictive pericarditis and constrictive pericarditis), which can cause significant compromise of cardiovascular function.

Prior to the advent of modern four-drug anti-tuberculous regimens, tuberculosis-related mortality was almost universal in patients with pericarditis. With the use of modern combination anti-tuberculous therapy, which is able to achieve pericardial sterilisation, prevent dissemination and cure tuberculous infection, the mortality rate had fallen to below 10% by the 1980s.

Given that anti-tuberculous therapy is now widely available and used almost empirically, the residual morbidity and mortality rate is driven predominantly by the second dreaded mechanism discussed above. Heart failure, haemodynamic collapse and death from reaccumulation of compressive effusion, constrictive pericarditis and effusive constrictive pericarditis occur in up to 30% of cases.^2^,^3^ Factors such as the size of effusion at presentation, whether or not patients underwent pericardiocentesis at onset, the degree of immunocompromise from HIV, and use of adjuvant therapies such as corticosteroids have all been shown, albeit inconsistently, to influence this composite complication rate in various studies.^3^,^6^

To date, corticosteroids remain the most studied adjuvant therapy to reduce the rates of post-tuberculous constriction and other compressive pericardial syndromes. In the largest of these studies, the IMPI trial, a six-week tapered course of prednisolone added to four-drug anti-tuberculous therapy reduced the incidence of pericardial constriction in both HIV-positive and -negative patients, compared to placebo, by approximately 50%.3 There has been much discussion and debate about the role of corticosteroids in this condition since the completion of the IMPI trial, particularly given the finding that HIV-infected patients had an increased risk of malignancy with the use of prednisolone.

With this background in mind, the study by Liebenberg et al., published in this issue of the journal (page 350), is a welcome attempt by colleagues who work in an environment where they are still confronted with the scourge of tuberculous pericarditis, to test whether an adjuvant medical intervention other than steroids may be effective. Drawing from the effectiveness of colchicine in preventing recurrences of acute idiopathic pericarditis, as demonstrated in the COPE trial,^7^ they sought to test whether the same agent added to standard anti-tuberculous therapy would significantly reduce the occurrence of pericardial constriction in patients with definite or probable tuberculous pericarditis, compared to placebo.

Thirty-three HIV-positive patients with definite or probable tuberculous pericarditis were randomly assigned to receive 1 mg per day of colchicine over six weeks (versus placebo) in addition to standard four-drug anti-tuberculous therapy for six months and oral steroids over eight weeks. Anti-retroviral therapy was available to all participants. Pericardial constriction was assessed by echocardiography after a follow-up period of 16 weeks in 21 participants. Five participants developed echocardiographic features of constriction with no demonstrable difference between the intervention and placebo arms of the study (relative risk 1.07, 95% CI: 0.46–2.46, p = 0.88).

While the focus of the authors’ conclusions and discussion centres around the absence of a significant efficacy outcome with colchicine, rather than despair about yet another negative study in TB pericarditis, it is important to keep in mind what this study actually tested and teaches us. The first lesson is about the importance of perseverance for clinician researchers. Just because steroids have been shown not to work, Liebenberg and colleagues have not folded their arms and given up trying to find an effective intervention to help their patients with this condition.

Their perseverance and that of others reminds us that there are in fact various other medical interventions that have the potential to be efficacious adjunctive therapies in tuberculous pericarditis but are as yet untested in large-scale clinical trials. The use of intra-pericardial fibrinolytic therapy to facilitate pericardial drainage is one such technique,^8^,^9^ which has been touted as potentially effective at reducing constriction by breaking up fibrous adhesions, and will be tested in the soon-to-be-launched second investigation into the management of pericarditis trial (IMPI-2). Angiotensin convertase enzyme inhibitors are capable of activating anti-fibrotic cytokines within the pericardium and may also hold promise.^10^,^11^ Finally, we should also applaud the on-going attempts to better understand the pathobiology of tuberculous pericarditis and its complications through highquality basic science research that will hopefully identify new targets for adjuvant therapies.^12^,^14^

Secondly, it is also important to emphasise that, strictly speaking, this study by Liebenberg et al. is by no means a negative study. Small pilot or vanguard studies such as this are not designed to provide definitive answers on efficacy; in fact, if they do, it’s likely that there is a type II or similar error. The main objectives of such studies, even if unstated, are actually to make sure that important design, method and safety issues necessary to successfully conduct an appropriately sized study are in place.

Important questions that often need addressing include: is the recruitment rate achieved in the pilot study adequate to reach calculated sample sizes in a reasonable time frame if a definitive study is conducted; is the investigational drug free of harmful side effects and safe enough in this unique condition to be tested on hundreds or thousands of patients; is the infrastructure and set up required to not lose any patients during follow up adequate; are the tools such as those for data collection and storage adequate; and finally, are the resources available and allocated for conducting a full study appropriate to ensure success? To this end the authors have gone some way in providing at least some of the required answers. On the face of it, a large, simple, randomised trial that is feasible and safe, and that would require multiple collaborating sites and mitigation strategies to prevent loss to follow up and completion within acceptable time frames is possible.

In conclusion, the authors should be congratulated for continuing to fight the good fight and demonstrating that we in the developing world must never lose sight of the principle that where there are unresolved clinical conditions unique to our environment, if we who face the problem do not try to find the solutions, no one else will do it for us. They have reminded us that it is imperative for us to continue to believe that there is no reason why with hard work, dedication and application, we cannot find appropriate solutions to improve the lot of our patients. We hope their demonstrated perseverance remains, and look forward to the news of a full-scale, large, randomised, controlled trial with them at the helm.
